# Asbestos-related cancer in naval personnel: findings from participants in the British nuclear tests 1952–1967

**DOI:** 10.1038/s41598-023-44847-4

**Published:** 2023-11-14

**Authors:** Richard T. Gun, Gerry M. Kendall

**Affiliations:** 1 School of Public Health, University of Adelaide, Adelaide, SA Australia; 2 Cancer Epidemiology Unit, Oxford Population Health, University of Oxford Old Road Campus, Oxford, OX3 7LF UK

**Keywords:** Cancer, Risk factors

## Abstract

Asbestos-containing materials (ACM) were present in British and Australian naval vessels throughout the twentieth century. The aim of this study was to identify and quantify the incidence of cancer in naval personnel from onboard asbestos exposure. Subjects were four cohorts of subjects who had served in the armed forces of the United Kingdom and Australia in the 1950s and 1960s. All cohorts had previously been studied, three of them in relation to radiation exposures from British nuclear testing. Comparisons of SIRs between services were made to identify cancers attributable to asbestos exposure. Excess mesotheliomas were found in naval personnel in all cohorts. In all but one cohort the lung cancer incidence was highest in navy personnel. Comparison of other smoking-related conditions indicated that the excess in navy personnel was not smoking-related. The relatively high SIRs for mesothelioma and the occurrence of deaths from asbestosis were indicative of high levels of asbestos exposure, with an expectation of cases of asbestos-related lung cancer. The findings are consistent with the occurrence of significant excesses of mesotheliomas. In addition, notwithstanding some inconsistencies in the results between the cohorts, we estimated that approximately 27% of lung cancers in Australian seamen and 12% in British seamen were related to onboard asbestos exposure.

## Introduction

Asbestos-containing materials (ACM) were present in British and Australian naval vessels throughout the twentieth century, and programs for asbestos elimination only began in the 1980s. Although the presence of ACM was widespread in naval vessels, the greatest likelihood of onboard exposure to airborne asbestos was to personnel working in engine rooms, where maintenance procedures involved disturbance of asbestos on pipework, turbines, boilers and other machinery. While general exhaust ventilation was provided in engine rooms (mainly for thermal comfort), safety measures which are now mandatory (e.g. local exhaust ventilation, masking of work areas, double locker rooms, supplied air respirators) did not apply at the time.

Several epidemiological studies of both naval and merchant mariners (summarised in Supplementary Table S1), have demonstrated excess mesotheliomas resulting from onboard asbestos exposure. There is less certainty as to the occurrence of asbestos-related lung cancers (ARLC). Excess lung cancer incidence or mortality has been found in nearly all studies, and while smoking data were mostly lacking, the incidence or mortality of other smoking-related cancers can indicate whether there is any unaccountable excess which could be attributed to occupational factors such as asbestos. Taking these factors into account, some studies have been suggestive of excess lung cancers from asbestos but most were inconclusive^1–9^. In the present context, a large mortality study of US nuclear test participants reported effects of asbestos exposure on mesothelioma and asbestosis in naval personnel, though a clear link with lung cancer was not established^9^.

While it is not possible to distinguish lung cancer cases caused by asbestos from those that are not, the likelihood of any ARLC cases may be estimated from cumulative exposure: coefficients of dose–response relationships have been generated for each of the asbestos fibre types and for mixed fibres^10^. High concentrations of airborne asbestos concentrations have been reported in British naval dockyards^11,12^, but no records of onboard asbestos exposure in British or Australian naval vessels have been located. Epidemiological studies have used proxies for exposure such as location (e.g. engine room) or duration of employment rather than actual asbestos exposure. In a review of asbestos-related cancer in naval personnel, Lemen and Landrigan cite asbestos exposures in US naval vessels orders of magnitude above permissible exposure limits during certain maintenance procedures, but no time-weighted average (TWA) exposure estimates were given^13^.

In the absence of exposure measurements, the likelihood and rate of occurrence of ARLCs in naval personnel may be inferred by comparing their mesothelioma incidence with those of other occupations. Gilham et al. have shown risks for both mesothelioma and ARLC incidence to be proportional to the asbestos fibre concentration in the lungs, and implicitly to cumulative exposure^14^. Therefore the highest proportion of lung cancers caused by asbestos are likely to be found in occupations with the highest mesothelioma incidence.

Another indicator of the likelihood of ARLC is the occurrence of asbestosis. There is evidence of a threshold exposure level of asbestos of 2 fibre/ml years, below which asbestosis does not occur^15^. Furthermore, a necropsy study of amphibole asbestos miners has shown asbestosis to be a significant risk factor for bronchial cancer, independent of cumulative fibre exposure^16^.

The aim of this study is to identify and, so far as is possible, to quantify any contribution from onboard asbestos exposure to the mesothelioma and lung cancer burden in British and Australian naval personnel.

## Material and methods

Most of the data presented herewith are derived from previously-published studies, as referenced.

The study population is comprised of four cohorts of Australian and British service personnel. Two cohorts are derived from British and Australian participants in the British atmospheric nuclear tests undertaken primarily in the 1950s (minor trials and clean-up operations continued into the 1960s). In the study of 21,357 British participants, cancer and mortality rates were compared with a control cohort of civilians and service personnel who had served overseas but not participated in the nuclear testing^17–20^. The control cohort contained a similar mix of subjects from the different armed services, and of officers and other ranks, as the cohort of test participants, and were also similar in matters such as date of birth^21^. The small percentage of civilians in each cohort were excluded from the present study.

A separate study of 8728 Australian test participants was completed in 2008^22–25^. Of this cohort 30% were civilians and were not included in the present study. There was no matched cohort of control subjects in the Australian study, but a contemporary cohort was available for comparison: Australian veterans of the Korean War^26,27^ (it was however not a fully independent cohort as 15% of the test participants had also served in Korea.)

The studies (other than of the Korean War veterans) were originally designed with a main objective of identifying any association between cancer incidence and radiation exposure, and we have summarised the effects of radiation in recent publications^28,29^. There was no indication of a link between lung cancer and radiation dose in the Australian^22,25^, British^17^, or US^9^ Studies.

In both the British and Australian studies the SIRs and SMRs were computed by comparison with the respective national male population, indirectly standardised by age and year of occurrence, using standard software programs. In computing confidence intervals both studies, observed cases were assumed to have a Poisson distribution.

For the present study, mortality and cancer data in naval veterans were compared with those of the army and the air force. To assess the contribution of smoking to lung cancer incidence, comparison of other smoking-related conditions was made by service. The analyses were based on tables from the published reports of the Australian and British nuclear test participants and of Australian Korean War veterans. Additional analyses (Table S2) were provided by the authors of the most recent update of the British study^17^. For the convenience of readers, approximate summaries of these data are also provided in which expected numbers were estimated by dividing observed numbers by the respective SIRs or SMRs.

Since the likelihood and incidence rate of ARLC is related to cumulative asbestos exposure and therefore to mesothelioma incidence, we compared SIRs of mesothelioma in naval veterans with high-risk occupations from two published studies: a study of mesothelioma mortality in Great Britain, in which risk was measured as proportional mortality ratio (PMR)^30^, and a study of mesothelioma incidence in Connecticut, in which the effect measure was Relative Risk, computed from Mantel–Haenszel odds ratios^31^.

Data on deaths from asbestosis by service were supplied by the authors of the update of the UK cohort study. Asbestosis deaths in the Australian cohort could not be ascertained as the data set is no longer readily available.

To estimate the number of lung cancers attributable to asbestos in naval veterans, we multiplied the expected number by the SIR for army veterans as an approximation for the number expected from smoking, and subtracted the product from the observed number in naval veterans.

### Ethics approval

The Human Research Ethics Committee of the University of Adelaide has authorised this project as exempt from requiring ethical review.

## Results

The number of subjects in each cohort, by service, and the percentage of officers in each are shown in Table 1.

**Table 1:** Number of subjects and percentage of officers in each cohort by service.

	Navy		Army		Air force	
	n	% officers	n	% officers	n	% officers
UK test participants	6305	7.7	5794	9.7	8443	19.1
UK control cohort	7343	7.9	5462	12.1	8702	20.7
Australian test participants	2613	9	1037	24	2459	22
Australian Korean War veterans	5102	NA	8934	NA	NA	NA

*NA* no data available.

Standardised Incidence Ratios (SIRs) for mesothelioma by service in the four cohorts are shown in Table 2. SIRs were elevated in naval veterans, and all elevations were statistically significant other than for the Australian Korean War veterans. Non-significant excesses were detected in British army veterans. SIRs were less than unity for Australian army veterans and all air force veterans.

**Table 2:** Standardised incidence ratios (SIRs) and 95% confidence intervals for mesothelioma in Australian and British nuclear test participants and control cohorts.

	Navy	Army	Air Force
Australian TP	2.79 (1.59–4.52)	0.98 (0.12–3.54)	0
Australian KWV	1.75 (0.83–2.67)	0.83 (0.34–1.32)	0
UK TP	2.62 (2.04–3.31)	1.18 (0.79–1.68)	0.49 (0.29–0.77)
UK controls	2.57 (2.04–3.20)	1.34 (0.93–1.86)	0.53 (0.32–0.82)

*TP* nuclear test participants, *KWV* Korean War veterans.

The SIRs for lung cancer are shown in Table 3. The SIR is higher in naval personnel than in the other armed services, with the exception of army veterans of the Korean War, in whom the SIR was the highest of all.

**Table 3:** Standardised incidence ratios (SIRs) and 95% confidence intervals for lung cancer in Australian and British nuclear test participants (TP) and control cohorts.

	Navy	Army	Air force
Australian TP	1.50 (1.26–1.77)	1.09 (0.78–1.49)	1.04 (0.84–1.28)
Australian KWV	1.25 (1.08–1.42)	1.59 (1.44–1.74)	0.82 (0.54–1.10)
UK TP	1.16 (1.05–1.27)	1.02 (0.91–1.14)	0.94 (0.86–1.03)
UK controls	1.15 (1.05–1.25)	1.01 (0.90–1.13)	0.82 (0.86–1.03)

To assess the possible contribution of smoking, estimates were made for other smoking-related conditions. The estimates from the British study as shown in Table 4 are for test participants and controls combined.

**Table 4:** Comparison between SIR for lung cancer and SIR for laryngeal cancer and standardised mortality ratio (SMR) for chronic obstructive pulmonary disease (COPD) by service, for both British cohorts combined.

	Navy		Army		Air Force	
	Obs	SIR/SMR	Obs	SIR/SMR	Obs	SIR/SMR
Lung cancer (SIR)	926	1.15	633	1.01	925	0.88
COPD (SMR)	418	0.93	313	0.97	410	0.67
IHD (SMR)	1934	0.92	1345	0.91	2129	0.76
Laryngeal cancer (SIR)	95	1.64	44	0.91	70	0.93
Bladder cancer (SIR)	269	1.00	217	1.05	316	0.91

Death rates from ischaemic heart disease (IHD) and chronic obstructive pulmonary disease (COPD) and incidence rates of bladder cancer in the navy and army are similar, suggesting that the excess lung cancers in the Naval veterans are not smoking-related. The SIR for laryngeal cancer is anomalous, being highest in the navy, and is discussed below. The estimates for the air force are all less than in the other services, suggesting a lower smoking prevalence than in the other services.

Similar comparisons in the Australian cohorts are shown in Table 5. For the test participants the SIRs for laryngeal and bladder cancer are higher in the army than the navy, suggesting that the higher SIR for lung cancer in the navy is not smoking-related (comparisons between the services for COPD and IHD mortality were not undertaken in the Australian study and the data sets are no longer readily available). As in the UK cohorts, the SIRs/SMRs are consistently less in the air force for all smoking-related conditions, again with the exception of bladder cancer.

**Table 5:** SIRs and SMRs of selected smoking-related conditions in Australian test participants and Korean War veterans.

	Navy		Army		Air Force	
	Obs	SIR/SMR	Obs	SIR/SMR	Obs	SIR/SMR
Nuclear test participants						
Lung cancer	138	1.50	39	1.09	94	1.04
Laryngeal cancer	16	1.51	7	1.82	13	1.35
Bladder cancer	30	1.07	13	1.14	37	1.29
Korean war veterans						
Lung cancer	203	1.25	435	1.59	32	0.82
Laryngeal cancer	27	1.40	65	2.05	3	0.71
Bladder cancer	63	1.31	85	1.03	14	1.16
IHD (SMR)	582	1.04	1230	1.18	139	0.84
COPD (SMR)	101	1.30	241	1.69	20	0.85

For the Korean veterans the mortality and incidence rates of smoking-related conditions are very high in army veterans. Even for lung cancer the SIR is higher in the army than in the navy. An exception is the lower SIR for bladder cancer in the army.

### Comparison of mesothelioma risk with other occupations.

In Table 6, SIRs for mesotheliomas in naval personnel are compared with all occupations in two other studies in which the risk was more than doubled (i.e. PMR or RR > 2).

**Table 6:** Mesothelioma SIRs in naval personnel compared with occupations with the highest ranked PMRs /RRs from other published studies.

Source	Occupation	Risk estimate	95% CI
Current study—naval personnel (SIR)	Australian TP	2.79	1.59–4.52
	Australian KWV	1.75	0.83–2.67
	UK TP	2.62	2.04–3.31
	UK controls	2.57	2.04–3.20
McElvenny et al. (PMR)^30^	Metal plate workers	5.02	4.44–5.65
	Vehicle body builders	5.26	4.19–6.52
	Plumbers and gas fitters	4.13	3.81–4.46
	Carpenters	3.88	3.62–4.13
	Electricians	2.79	2.55–3.04
	Electrical plant operators	2.63	1.97–3.43
	Electrical /electronic production fitters	2.60	1.71–3.78
	Sheet metal workers	2.35	1.98–2.73
	Chemical engineers and scientists	2.21	1.65–2.90
	Boiler operators	2.19	1.75–2.72
	Electrical engineers	2.16	1.81–2.53
	Construction workers nec	2.13	1.95–2.32
	Production fitters	2.09	1.96–2.24
Teta et al. (RR computed from Mantel–Haenszel odds ratio)^31^	Plumbers and pipefitters	3.87	1.38–10.82
	Engineers	2.72	0.86–8.68
	Cabinetmakers and carpenters	2.25	1.13–4.48
	Brickmasons and stonemasons	2.15	0.37–12.50

The SIRs in the naval personnel are shown to be comparable to those of the occupations ranked with the highest risk in both studies. Only four occupational categories in the UK study and two in the Connecticut study had higher SIRs for mesotheliomas.

### Asbestosis

In the UK cohort the number of deaths from asbestosis, for test participants and controls combined, was 12 in the navy (SMR 2.51), 2 in the army (SMR 0.51) and 4 in the air force (SMR (0.67).

### Ratio of ARLCs to mesotheliomas

Using the methodology described in the Material and Methods section, we estimated that about 50 ARLCs occurred in British test participants, and 60 in controls. The mesothelioma numbers were respectively 70 and 81, giving a ratio of lung cancers to mesotheliomas of 0.74 for both cohorts.

A considerably higher ratio of 2.4 was estimated for Australian test participants.

## Discussion

The high rates of mesothelioma seen only in naval veterans can be confidently attributed to onboard asbestos exposure, and are consistent with findings of other studies of naval and merchant seafarers, in particular in veterans of the US nuclear weapons testing^9^.

Lung cancer incidence was highest in the navy personnel and lowest in the air force, with the exception of the Korean War veterans’ cohort. The excess in naval personnel cannot be attributed automatically to asbestos: whereas most mesotheliomas are caused by asbestos, most lung cancers are not.

The largest excess of lung cancer in naval compared with army personnel is in Australian test participants, although the confidence intervals are wide: SIR 1.50 (1.26–1.77) in the navy vs 1.09 (0.78–1.49) in the army. While the difference in SIRs between the navy and the army is smaller in the British cohorts, the excess in the combined British cohorts is close to statistical significance: 1.15 (1.08–1.23) in the navy vs 1.01 (0.94–1.09) in the army (confidence intervals were estimated as (O/E)^1±1.96/chi^, where chi = (O − E)/√E).

It is likely that the low lung cancer incidence in the air force is at least partly due to a relatively high proportion of officers compared to other ranks. As shown in supplementary Table S2, officers have much lower lung cancer rates, whereas the proportion of officers in the navy and army cohorts are similar (Table 1). Comparisons between navy and army personnel are therefore not significantly confounded by rank (estimates of cancer SIR by rank were not undertaken in the Australian cohorts).

Comparisons of other smoking-related conditions showed that it is unlikely that the higher SIRs for lung cancer in naval personnel are due to higher smoking prevalence. Although in the British cohorts the SIRs for some conditions such as head and neck cancer and oesophageal and laryngeal cancer were higher in the navy, this may be alcohol-related: these cancers are related not only to smoking but to alcohol and an interactive combination of tobacco and alcohol^32–34^. The SMR for liver cirrhosis in naval veterans of 2.19 and 2.45 for test participants and controls respectively, compared with 0.87 and 1.27 in army veterans, suggest higher alcohol use in the navy (Table 4 and Table S4 in the supplement). On the other hand, for conditions such as COPD, ischaemic heart disease and bladder cancer which are related to smoking but not alcohol use, the data for both British and Australian services suggest that, if anything, smoking prevalence was higher in the army than in the navy (there are however some inconsistencies, such as a higher SIR for bladder cancer in the navy in the Korean War veterans cohort).

Evidence of a higher smoking prevalence in the British army is supported by a 1991 study of smoking in men in the three main branches of the British armed forces. A questionnaire survey by Lodge found that those in the army smoked more than those in the RN or RAF (41%, 36% and 26% current cigarette smokers respectively^35^. The data relate to a period well after the nuclear weapons tests, but it is plausible that differences in smoking patterns between the three armed forces have been persistent. Higher levels of smoking in army recruits were also reported by Bray ten years later^36^.

In the exceptional case of Australian veterans of the Korean War, the lung cancer SIR was higher in the army than the navy. It is therefore not possible to identify any contribution from asbestos to the lung cancer incidence in the navy, although we may conjecture that any effect of asbestos is obscured by negative confounding from an exceptionally high smoking prevalence in the Australian army personnel who served in the Korean War.

Tables of tobacco- and alcohol-related conditions for all cohorts are summarised in Tables S3–S5 in the supplement.

Since the lung cancer excesses in the navy are not explicable by higher smoking prevalence, asbestos is a likely alternative explanation. This is supported by the high SIRs for mesothelioma in naval veterans, comparable to those of occupations ranked with the highest levels of risk identified from other studies. High mesothelioma incidence indicates high cumulative asbestos exposure, with a corresponding high likelihood of some ARLCs.

This conclusion is supported by the occurrence of asbestosis deaths in the British navy, suggesting that the threshold exposure of 2fibre/ml years has been exceeded. The incremental risk of lung cancer for per fibre/ml year exposure to amphibole asbestos or mixed fibres has been estimated at 4.8%, so that the additional burden of lung cancer from asbestos exposure expected in an occupational group where asbestosis has occurred would be at least 10%^10^.

The low ratio of ARLCs to mesotheliomas in British naval personnel may reflect predominant exposure to crocidolite asbestos: in a review of 55 cohorts, McCormack and Peto found a mean of 0.7 ARLCs per mesothelioma for workers exposed to crocidolite, compared with ratios above unity for other fibres^37^. A report by Bartrip notes that by the end of World War 2, nearly all the major units of the British Fleet had their accommodation, engine rooms, and gun turrets insulated with sprayed limpet asbestos, a wet mixture of asbestos (usually crocidolite) water and cement^12^.

The higher ratio of 2.4 in Australian naval test participants suggests that the predominant exposure was to other species of asbestos.

These estimates equate to 27% of lung cancers in Australian seamen and 12% in British seamen being related to onboard asbestos exposure. The uncertainties in these estimates, which are substantial, include an assumption that absolute differences in observed cancers exactly equate to the number of ARLCs.

### Strengths and weaknesses

Apart from lack of data on smoking or asbestos exposure, the principal drawback is the absence of mortality data on asbestosis and of smoking-related conditions (other than cancer) in the Australian cohort. Unlike the British cohort, this study has not been updated and the data set is no longer readily available.

While the number of subjects in these cohorts is less than in comparable studies (see Supplementary Table S1), they are sufficient to derive stable estimates, as shown by the relatively narrow confidence intervals, especially for lung cancer.

The inference of a significant number of ARLCs in naval veterans, derived from the comparison with other service veterans, is strengthened by the finding of cases of asbestosis, which is a risk factor for lung cancer independent of exposure levels.

## Conclusion

Naval veterans have elevated rates of mesothelioma, not found in the other armed services, and attribution to onboard exposure to asbestos is non-contentious. They also have excess rates of lung cancer which are not fully explained by any differences in smoking prevalence.

The occurrence of mesothelioma is comparable to levels in occupational groups with the highest reported incidence or mortality from mesothelioma, suggesting that onboard exposures are comparable to those of industries with a high likelihood of ARLCs. Significant asbestos exposures are also indicated by the occurrence of asbestosis deaths in British naval veterans.

Notwithstanding some inconsistencies in the results between the cohorts, the findings are consistent with the occurrence not only of significant excesses of mesotheliomas but also of a substantial number of asbestos-related lung cancers.

### Supplementary Information

Supplementary Tables.

## Data Availability

The data presented in this paper are derived from previously-published studies. They can be accessed at the following websites: https://www.aihw.gov.au/getmedia/2cb2d58a-1d45-491a-ba2e-cb86b6a7e514/cis03.pdf.aspx?inline=true; https://www.dva.gov.au/sites/default/files/dosimetry_complete_study_1.pdf; https://www.dva.gov.au/sites/default/files/mortality_and_cancer_incidence_complete_study_1.pdf; https://www.aihw.gov.au/getmedia/3a5d6e95-2cf8-4dca-a384-b95b816bd263/Korean-Veterans-Mortality-Study.pdf.aspx?inline=true; https://iopscience.iop.org/article/10.1088/1361-6498/ac52b4.

## Abbreviations

ARLC: Asbestos-related lung cancer
SIR: Standardised incidence ratio
SMR: Standardised mortality ratio
IHD: Ischaemic heart disease
COPD: Chronic obstructive pulmonary disease
