# Supplementary material for: Asbestos-related cancer in naval personnel: findings from participants in the British nuclear tests 1952–1967
Source: Sci Rep. 2023 Nov 14;13:18842. doi: 10.1038/s41598-023-44847-4 (PMC10646006; doi:10.1038/s41598-023-44847-4)
Supplement: Supplementary file 1 — Supplementary Tables. [file 41598_2023_44847_MOESM1_ESM.docx]

**Asbestos-related cancer in naval personnel – findings from participants in the British nuclear tests 1952-1967**

**Supplementary tables**

| First author | Study type | Findings | Author comments |
| --- | --- | --- | --- |
| Rafnsson^1^ | Cohort of 6603 male marine engineers | Lung cancer SIR with 20-year lag time 1.3 (1.1-1.5). Non-significant excess pleural cancers, other tobacco cancers close to null . | The excess of lung cancer could be related to asbestos exposure. |
| Pukkala^2^ | 30940 male Finnish seafarers | Significant excess of mesothelioma. Significant excess of lung cancer only in engine room crews. | Occupational asbestos exposure among seafarers is likely strong enough to cause excess cases of mesothelioma but not of lung cancer |
| Saarni ^3^ | Nested case-control study of Finnish seafarers | Significant excess lung cancer in engine crew and deck officers. | Engine crews have an asbestos related risk of mesothelioma, and the engine room conditions also seem to increase risk of lung cancer. |
| Strand^4^ | 28,300 Norwegian naval personnel | Significant excess in engine crews of mesothelioma but not of lung cancer. | The mesothelioma incidence can  be taken as an indicator of the presence or absence of asbestos exposure, but it offered no  consistent explanation to the variation in incidence of other asbestos-related cancers. |
| Brandt^5^ | All merchant seamen identified from Danish census data | Excess respiratory cancer mortality in engine room crews and officers only. No excess mortality in these personnel from other smoking related diseases. | No conclusion stated on role of asbestos |
| Rapiti^6^ | 2208 merchant marine seamen in Italy | Significantly raised SMR from lung cancer in seamen who had sailed, increasing with employment duration | Increased risk of respiratory cancer among subjects with an occupational history of sailing; past exposure to asbestos and to other environmental carcinogens aboard could be implicated. |
| Petersen^7^ | 33084 Danish seafarers | Non-significant increase in mesothelioma incidence. Significant increase in incidence of lung cancer and other smoking-related cancers. | Asbestos exposure seems to affect the cancer pattern. |
| Petersen^8^ | 81740 male seafarers from the 5 Nordic countries | Significant excesses of mesotheliomas. Elevated lung cancer rates, but higher rates of other smoking-related cancers. | The majority of cancers could not be linked to specific occupational factors. |
| Boice^9^ | US nuclear test participants including 70309 naval personnel | Significant excess mesothelioma and lung cancer deaths in navy, but not other armed services.60 of the 65 asbestosis deaths were in the navy. | Significantly elevated SMRs for mesothelioma and  asbestosis, attributed to asbestos exposure aboard ships. |

**Table S1: Summary of epidemiological studies of respiratory cancer in naval and merchant seamen.**

|  | Disease | Officer participants | | Officer Controls | | Other rank participants | | Other rank controls | |
| --- | --- | --- | --- | --- | --- | --- | --- | --- | --- |
|  |  | Obs | SIR (95% CI) | Obs | SIR (95% CI) | Obs | SIR (95% CI) | Obs | SIR (95% CI) |
| RN | Buccal cavity and pharynx | 7 | 215 (86.6-443.8) | 6 | 151 (55.4-328.6) | 51 | 141* (104.7-184.9) | 84 | 198*** (158.2-245.5) |
|  | Oesophagus | 6 | 104 (38.3-226.9) | 6 | 84 (30.8-182.5) | 67 | 120 (92.8-152.0) | 82 | 126* (100.0-156.1) |
|  | Larynx | 1 | 40 (1.0-225.0) | 7 | 234 (94.1-482.4) | 39 | 161** (114.2-219.5) | 48 | 171*** (125.8-226.3) |
|  | Lung | 28 | 69* (45.7-99.4) | 17 | 34*** (20.1-55.1) | 403 | 121*** (109.8-133.8) | 478 | 125*** (113.9-136.5) |
|  | Pleura | 6 | 284* (104.3-618.7) | 7 | 261* (105.1-538.7) | 64 | 260*** (200.6-332.6) | 74 | 257*** (201.6-322.3) |
| Army | Buccal cavity and pharynx | 3 | 81 (16.7-236.2) | 6 | 136 (49.7-295.0) | 38 | 109 (77.4-150.2) | 27 | 79 (52.2-115.3) |
|  | Oesophagus | 5 | 75 (24.3-174.5) | 7 | 92 (36.8-188.6) | 42 | 85 (61.3-114.9) | 60 | 122 (93.0-156.9) |
|  | Larynx | 4 | 139 (37.7-354.7) | 1 | 31 (0.8-174.1) | 19 | 90 (54.5-141.3) | 20 | 96 (58.7-148.4) |
|  | Lung | 11 | 22*** (11.2-40.0) | 19 | 38*** (23.1-59.9) | 306 | 117** (104.0-130.6) | 297 | 113* (100.6-126.7) |
|  | Pleura | 0 | 0 (0-159.9) | 2 | 64 (7.7-230.7) | 30 | 130 (87.4-185.0) | 33 | 143 (98.6-201.2) |
| RAF | Buccal cavity and pharynx | 10 | 97 (46.7-179.3) | 20 | 178* (108.5-274.3) | 52 | 115 (86.2-151.3) | 51 | 111 (82.9-146.4) |
|  | Oesophagus | 13 | 71 (37.7-121.2) | 19 | 94 (56.4-146.2) | 65 | 97 (74.7-123.4) | 70 | 102 (79.6-129.1) |
|  | Larynx | 5 | 63 (20.6-148.0) | 6 | 68 (25.1-148.8) | 39 | 136 (96.7-185.9) | 20 | 68 (41.7-105.5) |
|  | Lung | 77 | 59*** (46.7-73.9) | 69 | 46*** (35.7-58.1) | 405 | 106 (95.7-116.6) | 374 | 95 (85.9-105.5) |
|  | Pleura | 1 | 15* (0.4-83.5) | 1 | 14* (0.4-80.7) | 17 | 56* (32.9-90.3) | 19 | 62* (37.2-96.6) |

**Table S2: Observed incident cancers (Obs), standardised incidence ratios (SIR) among test participants in the British nuclear tests and controls by rank and service for selected cancers. (Published with permission of M Gilles and R Haylock.)**

| Disease | Risk factor | Navy | | Army | | Air Force | |
| --- | --- | --- | --- | --- | --- | --- | --- |
|  |  | Obs | SIR/SMR | Obs | SIR/SMR | Obs | SIR/SMR |
| Head and neck cancer | Tobacco, alcohol, tobacco alcohol interaction | 148 | 1.72 | 74 | 0.96 | 133 | 1.18 |
| Oesophageal cancer | Tobacco, alcohol, tobacco alcohol interaction | 161 | 1.20 | 114 | 1.01 | 167 | 0.96 |
| Laryngeal cancer | Tobacco, tobacco alcohol interaction | 95 | 1.64 | 44 | 0.91 | 70 | 0.93 |
| Lung cancer | Tobacco | 926 | 1.15 | 633 | 1.01 | 925 | 0.88 |
| Bladder cancer | Tobacco | 269 | 1.00 | 217 | 1.05 | 316 | 0.91 |
| Ischaemic heart disease mortality | Tobacco | 1934 | 0.92 | 1345 | 0.91 | 2129 | 0.76 |
| COPD mortality | Tobacco | 418 | 0.93 | 313 | 0.97 | 410 | 0.67 |
| Cirrhosis mortality | Alcohol | 162 | 2.33 | 64 | 1.07 | 85 | 0.93 |

**Table S3: Incidence/mortality for tobacco- and alcohol-related diseases, for British test participants and controls combined.**

| Disease | Risk factor | Navy | | Army | | Air Force | |
| --- | --- | --- | --- | --- | --- | --- | --- |
|  |  | Obs | SIR | Obs | SIR | Obs | SIR |
| Head and neck cancer | Tobacco, alcohol, tobacco alcohol interaction | 43 | 1.48 | 16 | 1.49 | 35 | 1.31 |
| Oesophageal cancer | Tobacco, alcohol, tobacco alcohol interaction | 16 | 1.71 | 4 | 1.11 | 14 | 1.55 |
| Laryngeal cancer | Tobacco, tobacco alcohol interaction | 16 | 1.51 | 7 | 1.82 | 13 | 1.35 |
| Lung cancer | Tobacco | 138 | 1.50 | 39 | 1.09 | 94 | 1.04 |
| Bladder cancer | Tobacco | 30 | 1.07 | 13 | 1.14 | 37 | 1.29 |

**Table S4: Incidence of tobacco- and alcohol-related cancers in Australian test participants**

| Disease | Risk factor | Navy | | Army | | Air Force | |
| --- | --- | --- | --- | --- | --- | --- | --- |
|  |  | Obs | SIR/SMR | Obs | SIR/SMR | Obs | SIR/SMR |
| Head and neck cancer | Tobacco, alcohol, tobacco alcohol interaction | 68 | 1.93 | 110 | 1.91 | 12 | 1.61 |
| Oesophageal cancer | Tobacco, alcohol, tobacco alcohol interaction | 25 | 1.55 | 41 | 1.52 | 6 | 1.58 |
| Laryngeal cancer | Tobacco, tobacco alcohol interaction | 27 | 1.40 | 65 | 2.05 | 3 | 0.71 |
| Lung cancer | Tobacco | 203 | 1.25 | 435 | 1.59 | 32 | 0.82 |
| Bladder cancer | Tobacco | 63 | 1.31 | 85 | 1.03 | 14 | 1.16 |
| Ischaemic heart disease mortality | Tobacco | 582 | 1.04 | 1230 | 1.18 | 139 | 0.84 |
| COPD mortality | Tobacco | 101 | 1.30 | 241 | 1.69 | 20 | 0.85 |
| Cirrhosis mortaliy | Alcohol | 39 | 1.39 | 69 | 1.47 | 2 | 0.35 |

**Table S5: Incidence/mortality for tobacco- and alcohol-related diseases, for Australian veterans of the Korean War.**
